# The Preventive Effects of Diminazene Aceturate in Renal Ischemia/Reperfusion Injury in Male and Female Rats

**DOI:** 10.1155/2014/740647

**Published:** 2014-11-13

**Authors:** Maryam Malek, Mehdi Nematbakhsh

**Affiliations:** ^1^Water & Electrolytes Research Center, Department of Physiology, Isfahan University of Medical Sciences, Iran; ^2^Isfahan MN Institute of Basic & Applied Sciences Research, Isfahan, Iran

## Abstract

*Background*. Angiotensin-converting enzyme 2/angiotensin (1-7)/Mas receptor (ACE2/Ang-1-7/MasR) appears to counteract most of the deleterious actions of angiotensin-converting enzyme/angiotensin II/angiotensin II receptor 1 (ACE/Ang II/AT1R) in renal ischemia/reperfusion (I/R) injury but ACE2 activity and its levels are sexually dimorphic in the kidney. This study was designed to evaluate the effects of activation endogenous ACE2 using the diminazene aceturate (DIZE) in renal I/R injury in male and female rats. *Methods*. 36 Wistar rats were divided into two groups of male and female and each group distinct to three subgroups (*n* = 6). I/R group was subjected to 45 min of bilateral ischemia and 24 h of reperfusion, while treatment group received DIZE (15 mg/kg/day) for three days before the induction of I/R. The other group was assigned as the sham-operated group. *Results*. DIZE treatment in male rats caused a significant decrease in blood urea nitrogen (BUN), creatinine, liver functional indices, serum malondialdehyde (MDA), and increase kidney nitrite levels (*P* < 0.05), and in female rats a significant increase in creatinine and decrease serum nitrite levels compared to the I/R group (*P* < 0.05). *Conclusions*. DIZE may protect the male kidney from renal I/RI through antioxidant activity and elevation of circulating nitrite level.

## 1. Introduction

Renal ischemia/reperfusion (I/R) injury occurs in hypovolaemia conditions, septic shock, renal transplantation, or cardiovascular surgery, and it is the most common cause of acute kidney injury (AKI) which is associated with increased morbidity and mortality rates and subsequent development of chronic kidney disease (CKD) [1–3]. Evidences have shown that there is a protective effect of female sex hormone on I/R-induced AKI [4], but there is lower renal angiotensin-converting enzyme 2 (ACE2) level in the female than male rats [5]. Whereas there have been abundant pathophysiologic studies on renal I/R injury, a definitive treatment has not been found [6]. Several causal factors such as reactive oxygen species (ROS), cytokines, and chemokines synthesis contribute to the pathogenesis of renal damage [7, 8], among these mechanisms interaction between both arms of rennin angiotensin system (RAS), ACE-angiotensin II (Ang II)-Ang II receptor 1 (AT1R) and ACE2-angiotensin 1-7 (Ang 1-7)-Mas receptor (MasR) axis, has progressively assumed an important role [9, 10]. Many studies have shown that Ang II contributes to renal injury in AKI models [11, 12]. ACE2 is a homologue of ACE which cleaves a single amino acid from AngII and forms a heptapeptide with vasodilatory actions of Ang 1-7 playing a protective role in AKI [10]. Local balance between ACE and ACE2 as well as Ang II and Ang 1-7 is crucial for controlling AngII levels and its effects on renal injury; therefore ACE2 upregulators that can proper ACE/ACE2 balance are potential therapeutic strategy for kidney injury [13]. Diminazene aceturate (DIZE), an activator of endogenous ACE2, is widely used as a trypanolytic agent in livestock that can activate the protective axis of RAS and counter-regulates the deleterious effects of Ang II [14]. We hypothesized that intrinsic ACE2 activator such as DIZE would have protective effects on I/R-induced AKI, and to test these hypotheses male and female rats were subjected to I/R injury and the effect of DIZE was compared with control group.

## 2. Methods

### 2.1. Animals

36 Wistar rats were maintained at a room temperature of 23°C ± 2°C, with a 12 h light-dark cycle. Tap water and chow were freely available throughout the acclimatization and study periods. Experiments were carried out in accordance with Isfahan University of Medical Sciences Ethics Committee.

Animals were divided into two distinct groups of males and females (weighting 180 ± 30 g) and each group was divided into three subgroups (6 in each subgroup) as follows: group (1) I/R 45 min of bilateral renal ischemia followed by reperfusion, group (2) sham operation group; group (3) I/R 3 days pretreated with DIZE (Aburaihan Pharmaceutical Company, Tehran, Iran) (15 mg/kg) [15] group.

### 2.2. Surgical Procedures

The rats were anesthetized using 10 mg/kg of xylazine and 100 mg/kg of ketamine hydrochloride intraperitoneally [16]. Two small flank incisions were made and both kidneys were excited. The renal pedicles, containing the artery, vein, and nerve supplying each kidney, were carefully isolated and clamped for 45 minutes. Occlusion was verified visually by change in the color of the kidneys to a paler shade and reperfusion by a blush. So the animals without restoration blood flow were excluded from the experiment. In sham operation group rats underwent identical surgical procedures as I/R group without bilateral renal clamping. In group 3, rats were pretreated with injection of DIZE solution in distillated water (15 mg/kg/day) for 3 days. In 3rd day and 2 hours after the last injection, renal I/R were induced. Twenty-four hours after bilateral pedicle occlusion and reperfusion all rats were reanesthetized, blood samples were collected from the heart, and the serum was separated immediately, stored at −20°C until being assayed. Then animals were sacrificed by injection of potassium chloride (KCL 10%) into the heart; both kidneys and gonads were removed and weighed. The right kidneys were homogenized and centrifuged at 6000 g for 10 minutes; supernatant was centrifuged again at 15000 g for 2 minutes. The left kidneys were fixed in 10% formalin for histological analysis. All animals were weighed at the start and end of the experimental period.

### 2.3. Histological Examination

Formalin-fixed renal tissue was dehydrated, embedded in paraffin, and sliced into 4 *μ*m thick sections which were stained with hematoxylin and eosin. Histological lesion (acute tubular necrosis) was graded on a scale of 0 to 4 as follows [17]: 0 = normal kidney; 1 = minimal damage (<5% involvement of the cortex or outer medulla); 2 = mild damage (5–25% involvement of the cortex or outer medulla); 3 = moderate damage (25–75% involvement of the cortex or outer medulla); 4 = severe damage (>75% involvement of the cortex or outer medulla).

### 2.4. Laboratory Analysis

Serum creatinine, urea, alkaline phosphatase (ALP), aspartate aminotransferase (AST), and alanine aminotransferase (ALT) of all samples were measured using an autoanalyzer (Pars Azmun, Iran). Serum level of nitrite was assayed by using colorimetric assay kit (Promega Corporation, USA).

MDA levels in serum and kidney supernatant were assayed manually by the measurement of thiobarbituric acid-reactive substances (TBARS) levels at 532 nm, briefly 500 *μ*L of each sample was mixed to 10% trichloroacetic acid and centrifuged at 2000 g for 10 min, and 500 *μ*L of supernatant was added to 500 *μ*L of 0/67% thiobarbituric acid and incubated in warm water bath for 10 min.

### 2.5. Statistical Analysis

Data were expressed as the mean ± SEM. Differences between groups were compared by one-way analysis of variance using SPSS software version 20 for windows. Pathological damages scores between the groups were compared by Mann-Whitney, and *P* < 0.05 was considered statistically significant.

## 3. Results

### 3.1. Effect of I/R and Diminazene Treatment on Renal Function

**Figure 1:**
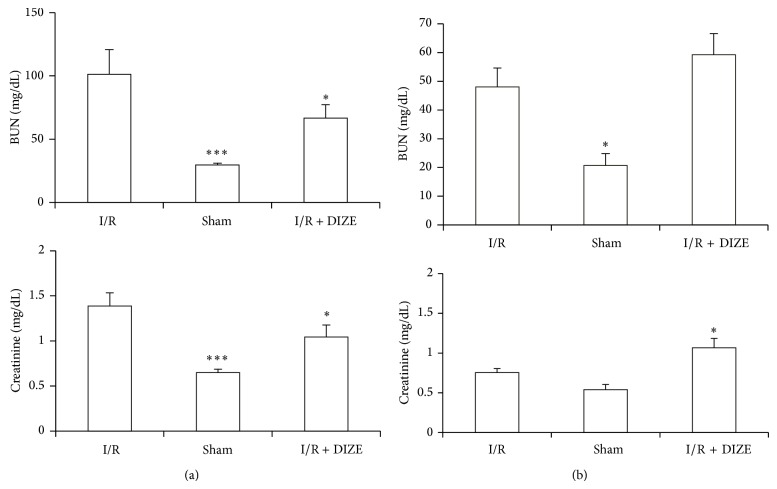
Changes in blood urea nitrogen (BUN) and creatinine levels in the male (a) and female (b) I/R (45 min ischemia followed by 24 hours of reperfusion), sham operated, and diminazene (15 mg/kg/day for 3 days) treated groups before ischemia (I/R + DIZE) (for each group *n* = 6).^*^ *P* < 0.05, ^***^ *P* < 0.001 versus I/R group.

There was an increase in BUN and creatinine levels following ischemia in both sexuality groups, but it showed only in male groups a significant increase when compared to the sham group (101.25 ± 19.5 versus 29.5 ± 1.42 mg/dL and 1.39 ± 0.14 versus 0.65 ± 0.037 mg/dL, *P* < 0.001). The elevation in BUN and creatinine levels in male groups was reduced significantly by DIZE pretreatment (66.74 ± 10.5 versus 101.25 ± 19.5 mg/dL and 1.04 ± 0.134 versus 1.39 ± 0.14 mg/dL); female treated group showed worsen response in BUN and creatinine levels with significant increase in creatinine level as compared to the I/R group (1.065 ± 0.118 versus 0.755 ± 0.049 mg/dL, *P* < 0.05) (Figures 1(a) and 1(b)).

### 3.2. Effect of Renal I/R and DIZE Treatment on Hepatic Function

**Figure 2:**
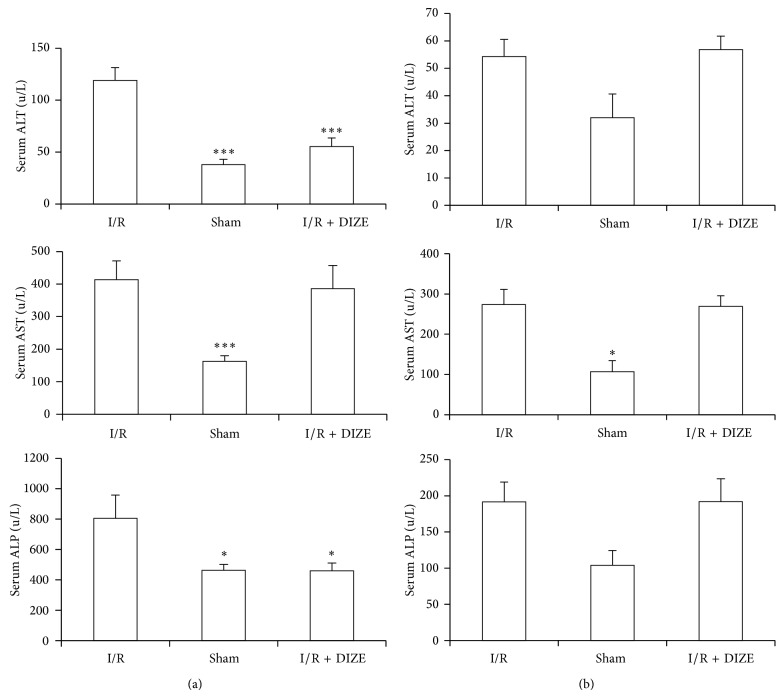
Changes in serum ALT, AST, and ALP in male (a) and female (b) IR (45 min ischemia followed by 24 hours of reperfusion), sham operated, and diminazene (15 mg/kg/day for 3 days) treated groups before ischemia (I/R + DIZE). ^*^ *P* < 0.05, ^***^ *P* < 0.001 versus I/R group.

As shown in Figures 2(a) and 2(b), serum levels of ALT, AST, and ALP elevated after ischemia in contrast to sham groups; however only male groups had significant difference (119 ± 12.24 versus 38 ± 4.9 u/L, 413.67 ± 57.86 versus 162.67 ± 16.82 u/L and 805.33 ± 153.87 versus 463.17 ± 39.2 u/L,  *P* < 0.05). DIZE administration before ischemia could decrease male serum levels of ALT and ALP when compared to IR groups (55.33 ± 8.19 versus 119 ± 12.24 u/L, and 460 ± 50.63 versus 805.33 ± 153.87 u/L, *P* < 0.05).

### 3.3. Effect of Renal I/R and DIZE Treatment on Serum and Kidney Levels of Nitrite

The serum nitrite level in female ischemia rats was higher significantly than the sham group (18.76 ± 2.58 versus 10.14 ± 0.72 *μ*mol/L, *P* < 0.05) and administration of DIZE attenuated it (13.08 ± 1.62 versus 18.76 ± 2.58 *μ*mol/L, *P* < 0.05). There was no significant difference in male rats.

**Figure 3:**
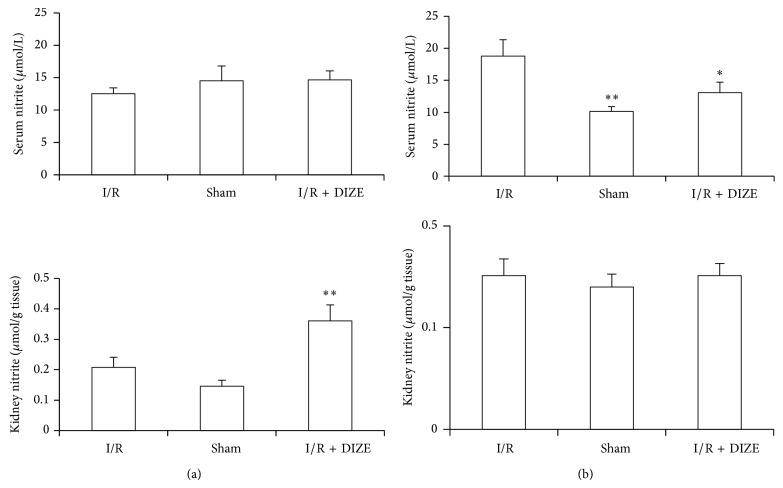
Changes in serum and kidney nitrate levels inmale (a) and female (b) I/R (45 min ischemia followed by 24 hours of reperfusion), sham operated, and diminazene (15 mg/kg/day for 3 days) treated groups before ischemia (I/R + DIZE). ^*^ *P* < 0.05, ^**^ *P* < 0.01 versus I/R group.

The kidney nitrite in male DIZE treated rats was elevated significantly when compared to I/R groups (0.36 ± 0.052 versus 0.208 ± 0.033 *μ*mol/g tissue, *P* < 0.05) but there was no significant difference in female rats (Figures 3(a) and 3(b)).

### 3.4. Effect of Renal I/R and DIZE Treatment on Gonads, Kidneys, and Body Weights

**Figure 4:**
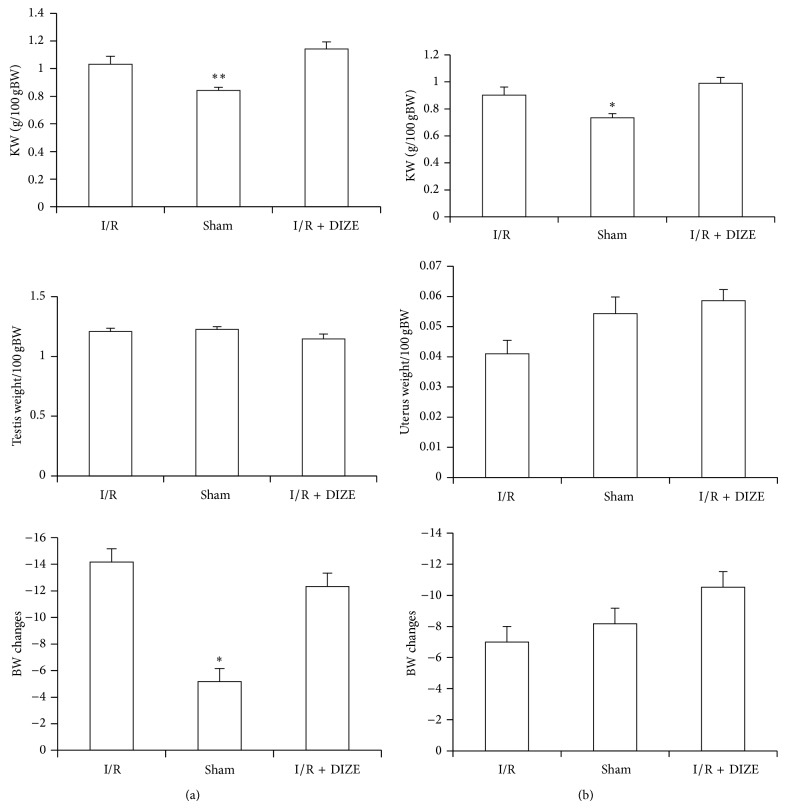
Total kidney weight (KW)/100 gr body weight, testis and uterus weight/100 gr body weight, and body weight changes in male (a) and female (b) I/R (45 min ischemia followed by 24 hours of reperfusion), sham operated, and diminazene (15 mg/kg/day for 3 days) treated groups before ischemia (I/R + DIZE). ^*^ *P* < 0.05, ^**^ *P* < 0.01 versus I/R group.

Body weight decreased significantly only in male ischemia compared to the sham group (*P* < 0.05). DIZE in female rats caused no more significant reduction in body weight (BW) than the ischemia group. After ischemia total kidney weight in both female and male rats was decreased significantly (*P* < 0.05) but DIZE had no effect on total kidney weight (TKW). Ischemia and DIZE had no effect on gonad (testis or uterus) weight (Figures 4(a) and 4(b).

### 3.5. Effect of Renal I/R and DIZE Treatment on Oxidative Stress

**Figure 5:**
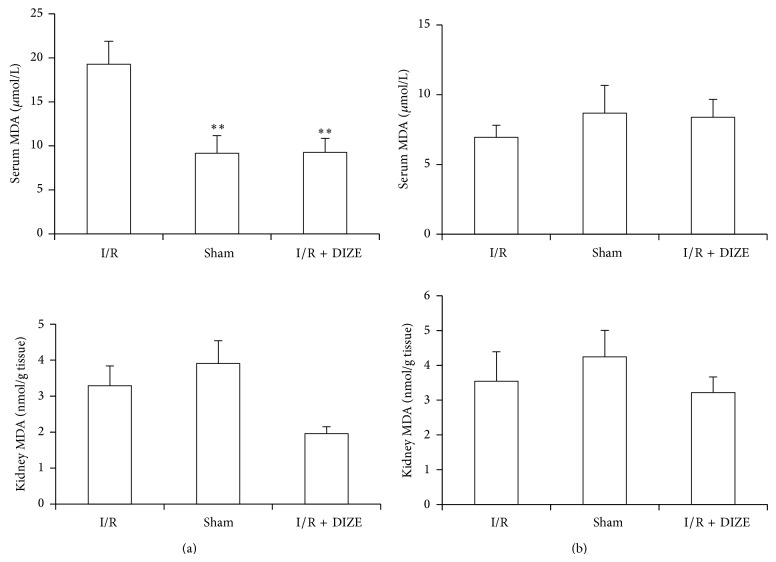
Changes in serum and kidney MDA levels in male (a) and female (b) I/R (45 min ischemia followed by 24 hours of reperfusion), sham operated, and diminazene (15 mg/kg/day for 3 days) treated groups before ischemia (I/R + DIZE). ^*^ *P* < 0.05, ^**^ *P* < 0.01 versus I/R group.

Ischemia and reperfusion caused only significant increase in male serum MDA levels compared to the sham group and DIZE treatment depressed it when compared to the IR group (19.27 ± 2.6 versus 9.15 ± 2.01 *μ*mol/L and 9.26 ± 1.6 versus 19.27 ± 2.6 *μ*mol/L, *P* < 0.05) (Figure 5(a)). Although administration of DIZE was decreased kidney MDA levels in male rats, but this difference was not statistically significant in compared to the ischemia group (Figures 5(a) and 5(b)).

### 3.6. Effect of Renal IR and DIZE Treatment on Kidney Histological Changes

**Figure 6:**
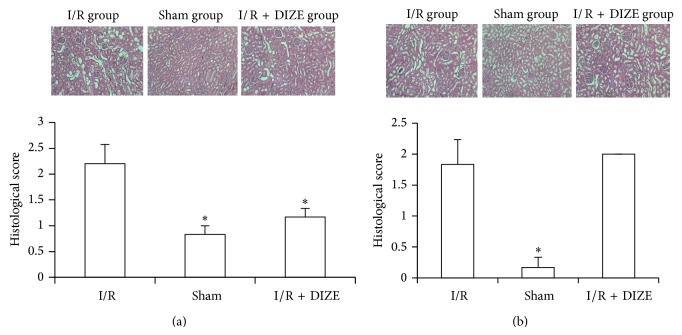
Histological evaluation of rat kidneys in male (a) and female (b) I/R (45 min ischemia followed by 24 hours of reperfusion), sham operated, and diminazene (15 mg/kg/day for 3 days) treated groups before ischemia (I/R + DIZE). ^*^ *P* < 0.05 versus I/R group.

The kidneys obtained from the I/R group (male and female rats) demonstrated obvious features of tubular damage compared to the sham group (*P* < 0.05) (Figure 6). Preischemic treatment with DIZE reduced significantly the extent and severity of injury in male rats compared to the I/R group (*P* < 0.05).

## 4. Discussion

The main objective of this research was to determine the effects of activation endogenous ACE2 using the DIZE in renal I/R injury. Several renoprotective anti-inflammatory effects of ACE2/Ang1-7/Mas receptor axis have been reported in kidney diseases suggesting that ACE2 may be playing role in kidney functions and renal hemodynamic regulation [18, 19]. ACE2 contributes to the balance of both RAS mediators, AngII and Ang1-7, because this enzyme can catabolize AngII to Ang1-7 which counteracts many pathophysiological effects of AngII [20]. Reduction of ACE2 expression or activity was observed in many acute kidney injures such as I/R which support important role of ACE2 in renal I/R [21, 22]. Previous studies demonstrated the elevation of AngII and reduction of Ang1-7 levels in I/R injured animals [21, 23, 24]. Administration of recombinant human ACE2 has been shown to mitigate injury in various disease models of elevated Ang II [25, 26]. Qi et al. showed that DIZE treatment attenuated ischemia-induced cardiac pathophysiology [15]. These findings encourage us to investigate the effect of DIZE, a drug recently described as an activator of ACE2, with anti-inflammatory effects [14, 27] in renal I/R injury. The elevation of serum urea and creatinine are the important early biomarkers in renal damages [28]. We demonstrated that DIZE was able to attenuate I/R-induced elevation of serum BUN and creatinine in male rats. The female rats displayed higher levels of creatinine compared to the I/R group that indicated sex difference responses of DIZE. It is widely accepted that apoptosis is the major pathological change in renal I/R injury. In histological observations; the I/R group in both male and female rats demonstrated obvious features of acute tubular damage compared to the sham group that these histological alterations were significantly attenuated only in male treated rats with DIZE compared with the ischemia group. These difference responses may be because ovarian hormones can downregulate ACE2 mRNA expression in the kidney that augmented reduction of renal I/R-induced ACE2 expression [18, 21, 29] and led to lower renal ACE2 level in the female than male rats [5]. Consequently lower compensatory mechanisms attenuate the extent of damaging effects of AngII. In addition, studies showed that the serum and kidney ACE2 activity is sex dependent with higher activity in males compared to females [30, 31]. Previous studies showed that administration of the Ang1-7 agonist induced significant renoprotection in male mice, with diminished serum creatinine, decreased histological injury, and reduced renal and pulmonary leukocyte infiltration in renal ischemia/reperfusion [32].

Lipid peroxidation is an important pathway of reactive oxygen species inducing tissue injury after ischemic acute renal failure [33]. MDA is a stable and easily measurable marker of lipid peroxidation [34, 35]. In this study the levels of kidney MDA were significantly increased in male I/R rats and DIZE treatment reversed the increase of MDA levels to a considerable extent near to sham group thereby confirming its antioxidant role in male I/R injury but not in female rats. Failure of other systems such as liver has shown followed by renal I/R [36]. Usually, the extent of hepatic damage is assessed by the increased level of cytoplasmic enzymes (ALT, AST, and ALP). In current study, increased serum hepatic enzymes levels after I/R were attenuated by DIZE treatment (significant decrease of ALT and ALP levels) in male rats, whereas there was the elevation of hepatic enzyme levels in I/R female rats compared to sham group, but only AST showed significant difference and DIZE could not decrease these enzymes.

Nitrite represents a circulating and tissue storage form of nitric oxide (NO) liberated specifically during ischemia and is highly selective therapy agent with cytoprotection effects on tissue ischemia/reperfusion injuries [37–39]. Feng et al. have shown that ACE2 overexpression in brain increases NO synthesizes (both eNOS and nNOS) [40]. In addition of NO-mediated cytoprotection, nitrite may act via NO-independent pathways [41, 42]. Nitrite has optimal intrinsic biological activity at low pH or deoxyhemoglobin conditions such as those that occurred in ischemia [43]. It was reported that NOS activity and nitrite formation in isolated proximal tubule cells were enhanced by 15 min of hypoxia followed reoxygenation [44]. We demonstrated that DIZE treatment in female rats inhibited the increase of serum nitrite level after I/R but in male rats caused higher increase of kidney nitrite level after I/R. Therefore the difference in plasma and kidney nitrite levels after I/R may be another mechanism of contrasting response of DIZE treatment.

However, as mentioned based on many previous studies such as Fang et al. study [45], we assumed that the mechanism of diminazene action was the activation of ACE2 but Haber et al. showed that the beneficial action of diminazene on Ang II-induced hypertension was ACE2 activity-independent [46]. Therefore, additional experiments will be required to better define the mechanism responsible for the protective effect of ACE2 in I/R.

## 5. Conclusions

Treatment with DIZE, an activator of ACE2, ameliorates male renal and liver functional impairment after renal I/R injury through enhancement nitrite levels and antioxidant activity but not in female rats, possibly due to gender differences in ACE2 activity and nitrite levels. Our results indicate that DIZE may be a novel therapeutic agent in male renal I/R injury.
